# An exploratory comparative analysis of kinematic characteristics in two elite Chinese show jumping riders

**DOI:** 10.3389/fpsyg.2026.1877384

**Published:** 2026-08-28

**Authors:** Xinyinan Wang, Deqing Wang, Haining Yu, Ruiji Liang, Lunbate Qiao, Wei Han

**Affiliations:** 1 Shandong Sport University, Jinan, China; 2 Huizhou Bojun Equestrian Sports Co., Ltd., Huizhou, China

**Keywords:** elite rider, obstacle height, rider kinematics, show jumping, three-dimensional kinematics

## Abstract

**Background:**

Three-dimensional rider kinematics across jumping phases and obstacle heights remain sparsely documented in elite Chinese show jumping. This exploratory case study examined angle-related kinematic characteristics in two elite Chinese riders under field-based jumping conditions.

**Methods:**

Three-dimensional video-based kinematic analysis was performed using the Simi Motion 3D system with direct linear transformation (DLT)-based camera calibration and three-dimensional reconstruction of selected anatomical landmarks. Two elite male riders rode the same experienced horse over the second vertical obstacle of a combination at 85, 105, and 140 cm. One successful trial per rider was analyzed at each obstacle-height condition. The approach, take-off, flight, and landing phases were segmented, and bilateral landmark-derived angle-related kinematic variables corresponding to the shoulder, hip, knee, and ankle regions were summarized using phase-averaged values and within-phase standard deviations, which represented temporal variability within a single trial. Between-rider differences in phase-averaged values were defined as Δmean = mean_A − mean_B. Only case-level descriptive analyses were performed; no inferential statistical tests were conducted.

**Results:**

Between-rider kinematic differences did not show a consistent monotonic increase across obstacle-height conditions. Instead, the measures showing larger between-rider differences shifted across obstacle-height conditions and jumping phases. During take-off, the right shoulder-related measure showed the clearest directional change, with Δmean values changing from −39.91° at 85 cm to −6.26° at 105 cm and +26.35° at 140 cm. During approach, hip-related differences showed a relatively consistent direction across conditions. During flight and landing, the measures showing larger differences shifted across body regions and involved both proximal and distal angle-related measures.

**Conclusion:**

These case-level findings indicate that the pattern and direction of between-rider differences varied across obstacle-height conditions and jumping phases. Because only two riders and one successful trial per rider at each condition were analyzed, the observed patterns cannot be assumed to represent stable rider-specific characteristics. Larger samples, repeated trials, quantitative horse-related measurements, and longitudinal designs are needed to examine the stability and practical relevance of these patterns.

## 1 Introduction

Equestrian show jumping requires the rider to continually adapt posture and limb position in coordination with the horse during approach, take-off, flight, and landing. Studies in equestrian biomechanics have linked rider posture, asymmetry, and horse–rider interaction with performance-related outcomes and horse welfare (Balog et al., 2026; Clayton et al., 2023). However, quantitative rider-centered evidence remains limited and fragmented (Balog et al., 2026).

In mounted jumping, the rider is part of the horse–rider system rather than an isolated moving body. Rider posture, symmetry, and body position can influence equine locomotion and thoracolumbar kinematics (Clayton et al., 2023; Egenvall et al., 2020; MacKechnie-Guire et al., 2020). Trunk kinematic differences have also been observed between riders with different experience levels (Clark et al., 2022). Rider pelvic roll and pitch have been quantified during ridden locomotion, further supporting the relevance of proximal postural variables in horse–rider biomechanics (Egenvall et al., 2022). These findings support interpreting rider kinematics within the horse–rider system rather than as isolated human movement.

Previous rider kinematic studies have examined specific aspects of rider position and movement in riding or jumping tasks. In competitive show jumping, rider position has been compared between elite and non-elite riders (Nankervis et al., 2015). Other work has examined trunk kinematics in simulator-based riding (Clark et al., 2022), rider kinematics and electromyographic activity during cross-country jumps (Fortier Guillaume et al., 2021), and horse–rider interaction during simulated jumping (Nemecek et al., 2018). These studies offer useful methodological and conceptual reference points, but they differ from the present work in rider population, task setting, simulator use, and jumping context. To our knowledge, direct quantitative evidence from elite Chinese show jumping riders during real jumping remains limited, particularly regarding angle-related kinematic measures across obstacle-height conditions and jumping phases. This gap is relevant because rider posture and limb position may vary across different jumping tasks and movement phases.

This exploratory case study used three-dimensional kinematic analysis to describe angle-related kinematic characteristics of the riders under real jumping conditions. The first objective was to describe between-rider differences in eight angle-related kinematic variables corresponding to the left and right shoulder, hip, knee, and ankle regions in two elite riders from the Chinese national show jumping team across three obstacle-height conditions (85, 105, and 140 cm) and four jumping phases (approach, take-off, flight, and landing). The second objective was to characterize the case-level kinematic profiles of the two riders. The third objective was to identify candidate observation points and testable hypotheses for subsequent rider-specific evaluation. Given the case-study design, the results were not intended to support population-level inference or general training recommendations for elite show jumping riders.

## 2 Materials and methods

### 2.1 Participants and experimental horse

Two male elite show jumping riders from the Chinese national show jumping team were recruited and coded as Rider A and Rider B. Both riders had extensive experience in high-level domestic and international competitions. Rider A was 41 years old, 175 cm in height, 75 kg in body mass, and had 26 years of training experience. Rider B was 41 years old, 168 cm in height, 72 kg in body mass, and had 21 years of training experience.

A 10-year-old Warmblood horse with a withers height of 172 cm was used in the experiment. The horse had long-term experience competing at the 140-cm level in show jumping. Both riders were familiar with the horse’s movement characteristics and rhythm. The same horse and training environment were used to reduce variability related to horse identity and testing context across riders and obstacle-height conditions. However, the use of a single horse could not eliminate potential horse-related confounding.

Before formal testing, both riders completed a standardized warm-up consisting of walk, trot, canter, and low-obstacle jumping to reduce injury risk for both horse and rider. The obstacle heights were tested in ascending order: 85 cm, 105 cm, and 140 cm. One successful jump trial per rider was analyzed at each obstacle-height condition. A successful trial was defined as a jump completed without refusal, rail displacement, obvious loss of balance, interruption of the approach–take-off–flight–landing sequence, or camera occlusion that prevented key-point digitization.

### 2.2 Methods

#### 2.2.1 Fixed-position, fixed-focus video recording

Three fixed-position, fixed-focus high-definition video cameras were used for data collection (Sony HXR-NX200; 1,920 × 1,080 pixels; 50 Hz; shutter speed: 1/600 s). A three-dimensional calibration frame was placed in front of the second vertical obstacle of the combination. Spatial camera calibration was performed using the direct linear transformation (DLT) method to obtain calibration parameters for three-dimensional reconstruction (Abdel-Aziz and Karara, 2015). Before data collection, the calibration-point layout and camera geometry were checked to confirm that the calibrated volume covered the approach-to-landing movement area and that the camera views overlapped sufficiently.

**Figure 1:**
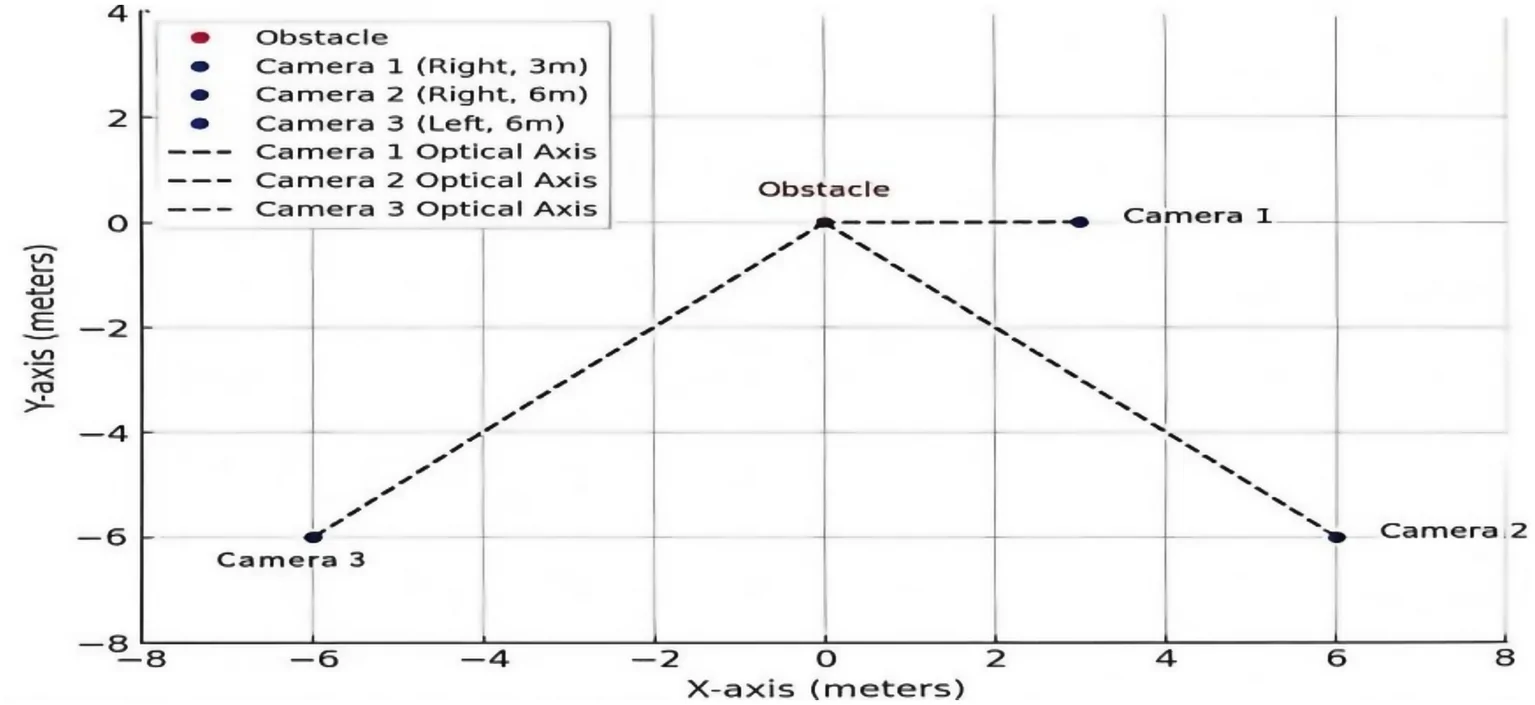
Schematic illustration of the obstacle position, camera locations, and optical axes used for multi-view video recording.

Camera 1 was positioned approximately 3 m to the right of the obstacle center, whereas Cameras 2 and 3 were positioned approximately 6 m in front of the obstacle on the right and left sides, respectively. The optical axes of the three cameras were directed toward the center of the obstacle area, with approximate inter-camera angles of 45° between Cameras 1 and 2 and 90° between Cameras 2 and 3. This camera geometry was intended to improve multi-view coverage of the rider during the approach-to-landing sequence and reduce visual occlusion during key-point digitization. The three cameras were synchronized using a light-signal synchronizer. Frame-level alignment was based on the LED flash that appeared in corresponding frames in the three camera views. The calibrated recording region covered the area surrounding the second vertical obstacle. Figure 1 shows the relative positions of the obstacle and the three cameras, as well as the direction of the optical axes used for multi-view recording.

Three obstacle-height conditions—85 cm, 105 cm, and 140 cm—were included in the analysis. One successful trial per rider was analyzed at each obstacle-height condition, resulting in six analyzed jump trials in total (2 riders × 3 heights). Data collection was conducted in a standard show jumping arena with a fiber-sand surface. The ambient temperature during data collection was approximately 25 ± 2 °C.

#### 2.2.2 Three-dimensional kinematic analysis

Three-dimensional kinematic analysis of the recorded videos was performed using the Simi Motion 3D system (Simi Reality Motion Systems, Germany), an image-based platform for motion capture and 2D/3D movement analysis. The three-dimensional coordinates of the selected key points were reconstructed from the synchronized multi-camera videos using the DLT-based spatial calibration parameters.

A trained operator performed frame-by-frame digitization of visible anatomical landmarks for each rider. Among the digitized landmarks, those corresponding to the left and right shoulder, hip, knee, and ankle regions were selected *a priori* for the present rider-centered kinematic analysis because these regions were considered biomechanically relevant to rider postural organization during the approach, take-off, flight, and landing phases of show jumping. This focused selection was intended to characterize posture-related kinematic patterns in selected body regions rather than to provide a whole-body biomechanical model of the rider. The resulting shoulder-, hip-, knee-, and ankle-related measures were treated as landmark-derived angle-related kinematic variables and interpreted as descriptive kinematic indicators rather than direct anatomical joint-angle measurements.

To reduce high-frequency noise in the coordinate and angle-related time series, a low-pass smoothing procedure was applied. The reconstructed trajectories were then visually inspected, and obvious digitization errors or outliers were checked frame by frame and corrected when necessary. However, neither intra-rater nor inter-rater digitization reliability was quantitatively assessed. Accordingly, although visual inspection and frame-by-frame correction were used as quality-control procedures, operator-related digitization variability could not be formally quantified. Quantitative indicators of DLT reconstruction quality, such as calibration accuracy or reconstruction error, were not independently quantified in the present study. Therefore, the measurement precision of the reconstructed coordinates and derived angle-related kinematic variables could not be fully evaluated, and these values should be interpreted with caution within the exploratory scope of the study.

The jumping action was divided frame by frame into four phases according to the horse’s movement relative to the obstacle. The approach phase was defined as the final three strides before take-off. The take-off phase was defined as the period from forelimb lift-off to the point at which the horse became fully airborne. The flight phase was defined as the period from full suspension to immediately before the first forelimb contact after the obstacle. The landing phase was defined as the period from initial forelimb contact to the completion of hindlimb landing and impact absorption.

#### 2.2.3 Statistical analysis

All data processing and descriptive statistical analyses were performed using Microsoft Excel 2021 and SPSS 26.0 (IBM Corp., Armonk, NY, USA). First, frame-level data corresponding to the different jumping phases were organized and classified in Excel. For each rider, the mean and standard deviation (SD) were calculated for each angle-related kinematic variable within each obstacle-height condition and jumping phase. The SD was calculated as the sample standard deviation and was used descriptively to indicate within-phase temporal variability of the angle-related time series within a single jump trial.

In addition, the between-rider difference in each angle-related measure was calculated under the same obstacle-height condition and jumping phase. This difference was defined as Δmean = mean_A − mean_B, where mean_A and mean_B represent the phase-averaged values of the corresponding angle-related measure for Rider A and Rider B, respectively. Δmean was used to describe the direction and magnitude of the observed between-rider kinematic differences.

Given the exploratory case-study design, the inclusion of only two riders, and the analysis of only one successful jump trial per rider at each obstacle-height condition, there were insufficient independent repeated measurement units to support inferential statistical analysis. In addition, the frame-by-frame data within each phase were temporally correlated and therefore could not be treated as independent observations. Accordingly, no between-rider significance testing was performed, and no *p* values, confidence intervals, or group-level effect sizes were reported. Descriptive statistics were used only to present the case-level kinematic characteristics and between-rider differences observed across the obstacle-height conditions and jumping phases.

#### 2.2.4 Ethics statement

This study was approved by the Sports Science Ethics Committee of Shandong Sport University (approval number: 2023045). Both riders were fully informed of the study purpose, procedures, and potential risks and provided written informed consent before participation. All experimental procedures were conducted under routine training conditions and did not interfere with the riders’ normal training. The procedures involving the horse were non-invasive and were conducted as part of routine show jumping training. No additional clinical intervention was performed.

## 3 Results

**Figure 2:**
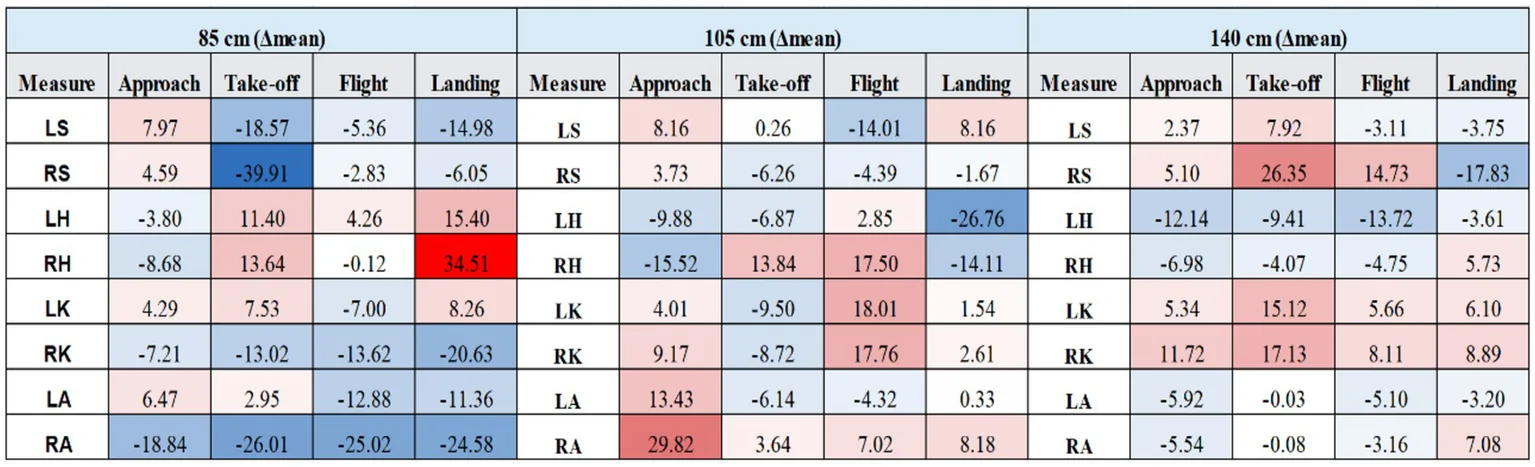
Obstacle-height-by-phase heatmaps of between-rider differences in angle-related kinematic measures. Each cell represents the phase-averaged between-rider difference for a specific angle-related measure and jumping phase (Δmean = phase-averaged value for Rider A − phase-averaged value for Rider B). Panels correspond to obstacle-height conditions of 85, 105, and 140 cm. Red indicates larger values in Rider A, whereas blue indicates larger values in Rider B. The color scale was fixed across panels from −40° to 40°.

This study descriptively compared angle-related kinematic measures corresponding to the shoulder, hip, knee, and ankle regions in two elite riders during one successful trial per rider over the second vertical obstacle of a combination at each of three obstacle-height conditions: 85 cm, 105 cm, and 140 cm (Tables 1–3; Figure 2). The jumping action was analyzed across four phases: approach, take-off, flight, and landing. All measures are presented as the mean ± standard deviation of frame-level values within each phase. The standard deviation reflects temporal variability within the corresponding phase of a single jump trial and does not represent between-trial variability or movement repeatability. Between-rider differences were expressed as the difference in phase-averaged values and were defined as Δmean = mean_A − mean_B. Given the limited sample size and number of trials, the findings are presented only as case-level kinematic descriptions across obstacle-height conditions and jumping phases. No population-level inference or significance testing was performed.

**Table 1:** Comparison of angle-related kinematic measures between the two riders over the second vertical obstacle of the combination at an obstacle height of 85 cm.

Phase/measure	Rider A (°)	Rider B (°)	Δmean = mean_A − mean_B
Approach			
LS	30.96 ± 16.00	22.99 ± 12.17	7.97
RS	23.37 ± 11.53	18.78 ± 9.31	4.59
LH	118.84 ± 8.75	122.64 ± 19.86	−3.80
RH	118.60 ± 8.75	127.28 ± 18.47	−8.68
LK	123.01 ± 7.70	118.72 ± 15.27	4.29
RK	122.09 ± 7.33	129.30 ± 14.99	−7.21
LA	101.49 ± 8.85	95.02 ± 8.15	6.47
RA	89.28 ± 8.09	108.12 ± 9.28	−18.84
Take-off			
LS	15.42 ± 3.69	33.99 ± 3.74	−18.57
RS	14.02 ± 6.55	53.93 ± 16.55	−39.91
LH	123.87 ± 6.26	112.47 ± 6.02	11.40
RH	133.98 ± 10.16	120.34 ± 7.15	13.64
LK	128.73 ± 4.43	121.20 ± 4.46	7.53
RK	121.10 ± 4.92	134.12 ± 4.74	−13.02
LA	107.85 ± 3.35	104.90 ± 4.40	2.95
RA	96.45 ± 8.77	122.46 ± 6.67	−26.01
Flight			
LS	25.53 ± 10.40	30.89 ± 4.36	−5.36
RS	19.33 ± 6.71	22.16 ± 2.61	−2.83
LH	118.57 ± 7.03	114.31 ± 10.75	4.26
RH	121.32 ± 12.01	121.44 ± 9.89	−0.12
LK	124.12 ± 10.31	131.12 ± 5.32	−7.00
RK	124.12 ± 14.19	137.74 ± 5.26	−13.62
LA	100.86 ± 6.34	113.74 ± 7.06	−12.88
RA	104.65 ± 9.98	129.67 ± 5.42	−25.02
Landing			
LS	18.94 ± 1.71	33.92 ± 4.65	−14.98
RS	17.82 ± 2.64	23.87 ± 1.15	−6.05
LH	112.49 ± 6.14	97.09 ± 8.39	15.40
RH	138.65 ± 4.39	104.14 ± 7.54	34.51
LK	133.27 ± 6.42	125.01 ± 3.78	8.26
RK	121.28 ± 8.59	141.91 ± 2.71	−20.63
LA	102.90 ± 11.16	114.26 ± 8.26	−11.36
RA	99.14 ± 8.22	123.72 ± 7.64	−24.58

LS, left shoulder-related measure; RS, right shoulder-related measure; LH, left hip-related measure; RH, right hip-related measure; LK, left knee-related measure; RK, right knee-related measure; LA, left ankle-related measure; RA, right ankle-related measure. Δmean was calculated as the phase-averaged value of the corresponding angle-related measure for Rider A minus that for Rider B (A − B, °). Negative values indicate larger values of the corresponding measure in Rider B, whereas positive values indicate larger values of the corresponding measure in Rider A. SD represents the sample standard deviation of the frame-level values and describes temporal variability of the corresponding angle-related measure within the corresponding phase of a single jump trial.

**Table 2:** Comparison of angle-related kinematic measures between the two riders over the second vertical obstacle of the combination at an obstacle height of 105 cm.

Phase/measure	Rider A (°)	Rider B (°)	Δmean = mean_A − mean_B
Approach			
LS	34.77 ± 16.36	26.61 ± 7.13	8.16
RS	28.47 ± 22.05	24.74 ± 4.22	3.73
LH	128.98 ± 13.56	138.86 ± 8.86	−9.88
RH	124.49 ± 13.24	140.01 ± 9.31	−15.52
LK	134.48 ± 10.17	130.47 ± 12.51	4.01
RK	141.60 ± 11.33	132.43 ± 13.68	9.17
LA	115.69 ± 10.15	102.26 ± 12.40	13.43
RA	126.10 ± 11.33	96.28 ± 11.71	29.82
Take-off			
LS	32.06 ± 3.28	31.80 ± 7.00	0.26
RS	23.31 ± 3.48	29.57 ± 6.63	−6.26
LH	113.49 ± 4.45	120.36 ± 5.95	−6.87
RH	132.64 ± 5.48	118.80 ± 6.87	13.84
LK	129.88 ± 3.47	139.38 ± 8.84	−9.50
RK	138.02 ± 6.90	146.74 ± 8.34	−8.72
LA	107.74 ± 3.41	113.88 ± 6.32	−6.14
RA	117.09 ± 9.14	113.45 ± 7.65	3.64
Flight			
LS	23.49 ± 4.08	37.50 ± 3.75	−14.01
RS	29.75 ± 6.75	34.14 ± 1.53	−4.39
LH	119.01 ± 8.16	116.16 ± 4.55	2.85
RH	137.11 ± 9.29	119.61 ± 10.34	17.50
LK	137.94 ± 6.64	119.93 ± 8.04	18.01
RK	150.33 ± 6.56	132.57 ± 8.91	17.76
LA	108.50 ± 12.89	112.82 ± 6.12	−4.32
RA	113.69 ± 5.02	106.67 ± 4.15	7.02
Landing			
LS	38.26 ± 6.24	30.10 ± 4.53	8.16
RS	22.14 ± 5.49	23.81 ± 4.75	−1.67
LH	97.26 ± 2.19	124.02 ± 9.31	−26.76
RH	119.91 ± 4.44	134.02 ± 8.43	−14.11
LK	127.65 ± 3.67	126.11 ± 6.97	1.54
RK	138.66 ± 2.35	136.05 ± 11.57	2.61
LA	114.64 ± 3.17	114.31 ± 6.72	0.33
RA	127.02 ± 7.10	118.84 ± 11.61	8.18

LS, left shoulder-related measure; RS, right shoulder-related measure; LH, left hip-related measure; RH, right hip-related measure; LK, left knee-related measure; RK, right knee-related measure; LA, left ankle-related measure; RA, right ankle-related measure. Δmean was calculated as the phase-averaged value of the corresponding angle-related measure for Rider A minus that for Rider B (A − B, °). Negative values indicate larger values of the corresponding measure in Rider B, whereas positive values indicate larger values of the corresponding measure in Rider A. SD represents the sample standard deviation of the frame-level values and describes temporal variability of the corresponding angle-related measure within the corresponding phase of a single jump trial.

**Table 3:** Comparison of angle-related kinematic measures between the two riders over the second vertical obstacle of the combination at an obstacle height of 140 cm.

Phase/measure	Rider A (°)	Rider B (°)	Δmean = mean_A − mean_B
Approach			
LS	27.51 ± 17.77	25.14 ± 7.14	2.37
RS	26.65 ± 9.30	21.55 ± 4.49	5.10
LH	114.59 ± 15.37	126.73 ± 15.38	−12.14
RH	129.75 ± 13.37	136.73 ± 10.11	−6.98
LK	132.28 ± 9.98	126.94 ± 14.05	5.34
RK	136.67 ± 9.77	124.95 ± 13.48	11.72
LA	116.17 ± 10.12	122.09 ± 6.77	−5.92
RA	110.90 ± 7.63	116.44 ± 8.68	−5.54
Take-off			
LS	41.89 ± 2.68	33.97 ± 3.65	7.92
RS	44.44 ± 7.89	18.09 ± 6.14	26.35
LH	100.24 ± 6.01	109.65 ± 4.35	−9.41
RH	122.60 ± 2.81	126.67 ± 6.18	−4.07
LK	131.85 ± 2.64	116.73 ± 4.21	15.12
RK	141.81 ± 1.98	124.68 ± 7.32	17.13
LA	122.24 ± 2.20	122.27 ± 3.52	−0.03
RA	114.92 ± 4.69	115.00 ± 5.64	−0.08
Flight			
LS	26.83 ± 8.39	29.94 ± 3.42	−3.11
RS	37.71 ± 8.63	22.98 ± 5.86	14.73
LH	119.90 ± 6.68	133.62 ± 14.38	−13.72
RH	136.35 ± 4.80	141.10 ± 8.91	−4.75
LK	140.77 ± 8.04	135.11 ± 15.42	5.66
RK	148.63 ± 6.02	140.52 ± 7.09	8.11
LA	127.94 ± 4.12	133.04 ± 4.60	−5.10
RA	123.42 ± 2.51	126.58 ± 4.01	−3.16
Landing			
LS	35.60 ± 12.26	39.35 ± 2.52	−3.75
RS	26.30 ± 2.83	44.13 ± 4.99	−17.83
LH	99.21 ± 5.80	102.82 ± 7.04	−3.61
RH	112.29 ± 6.32	106.56 ± 7.10	5.73
LK	124.55 ± 3.88	118.45 ± 3.38	6.10
RK	133.42 ± 5.03	124.53 ± 4.97	8.89
LA	122.64 ± 7.63	125.84 ± 5.14	−3.20
RA	126.35 ± 6.31	119.27 ± 11.84	7.08

LS, left shoulder-related measure; RS, right shoulder-related measure; LH, left hip-related measure; RH, right hip-related measure; LK, left knee-related measure; RK, right knee-related measure; LA, left ankle-related measure; RA, right ankle-related measure. Δmean was calculated as the phase-averaged value of the corresponding angle-related measure for Rider A minus that for Rider B (A − B, °). Negative values indicate larger values of the corresponding measure in Rider B, whereas positive values indicate larger values of the corresponding measure in Rider A. SD represents the sample standard deviation of the frame-level values and describes temporal variability of the corresponding angle-related measure within the corresponding phase of a single jump trial.

### 3.1 Overall kinematic patterns across obstacle-height conditions

Across the three obstacle-height conditions, the magnitude and direction of between-rider differences varied across jumping phases and angle-related measures, with no consistent monotonic increase across conditions.

At 85 cm, larger between-rider differences were observed primarily from take-off to landing, although the right ankle-related measure also showed a difference of Δmean = −18.84° during approach. At take-off, the largest absolute difference was observed in the right shoulder-related measure (Δmean = −39.91°), accompanied by a right ankle-related difference of Δmean = −26.01°. In flight, the larger differences involved the right knee-related measure and bilateral ankle-related measures, with the right ankle-related measure showing the largest absolute difference (Δmean = −25.02°). During landing, the larger absolute differences involved both proximal and distal body regions, including the right hip-, right ankle-, right knee-, left hip-, and left shoulder-related measures.

At 105 cm, between-rider differences were observed across all four jumping phases and multiple angle-related measures. During approach, the hip-related differences had the same direction, with Rider B showing larger values in both hip-related measures than Rider A. The ankle-related measures showed the opposite pattern, especially the right ankle-related measure (Δmean = 29.82°). At take-off, the larger differences involved the right hip- and bilateral knee-related measures. In flight, the larger differences involved the bilateral knee-related measures and the right hip-related measure, with an additional difference in the left shoulder-related measure. During landing, the left hip-related measure had the largest absolute between-rider difference at this condition (Δmean = −26.76°), accompanied by a right hip-related difference.

At 140 cm, the larger between-rider differences involved shoulder-, hip-, and knee-related measures, with both knee-related measures showing their largest absolute between-rider differences during take-off. At take-off, the between-rider difference in the right shoulder-related measure changed sign, becoming positive at 140 cm (Δmean = 26.35°) after being negative at 85 and 105 cm. In flight, the larger differences involved the right shoulder- and left hip-related measures. During landing, the right shoulder-related measure had the largest absolute difference at this condition (Δmean = −17.83°). All other landing-phase differences at 140 cm had absolute Δmean values below 10°.

### 3.2 Phase-specific between-rider kinematic patterns during jumping

When the four jumping phases were considered separately, the larger between-rider differences varied across obstacle-height conditions and angle-related measures within each phase.

During the approach phase, the larger differences often involved the hip- and ankle-related measures, although the pattern varied across obstacle-height conditions. The hip-related differences followed a consistent direction across the three conditions, with Rider B showing larger values in both hip-related measures than Rider A. The between-rider differences in both ankle-related measures showed sign changes across obstacle-height conditions. In particular, the between-rider difference in the right ankle-related measure changed from −18.84° at 85 cm to +29.82° at 105 cm and −5.54° at 140 cm.

During the take-off phase, the between-rider difference in the right shoulder-related measure showed a directional change across obstacle-height conditions. It changed from −39.91° at 85 cm to −6.26° at 105 cm and +26.35° at 140 cm. Hip-related differences also changed direction across obstacle-height conditions, whereas the bilateral knee-related measures showed their largest absolute between-rider differences at 140 cm. Thus, take-off differences were not concentrated in a single body region but shifted across shoulder-, hip-, and knee-related measures across obstacle-height conditions.

During the flight phase, the body regions with larger between-rider differences changed across obstacle-height conditions. At 85 cm, these differences were mainly found in the right knee-related measure and bilateral ankle-related measures, with the right ankle-related measure showing the largest absolute difference (Δmean = −25.02°). At 105 cm, the larger differences involved the bilateral knee-related measures and the right hip-related measure, with an additional left shoulder-related difference. At 140 cm, the larger differences involved the right shoulder- and left hip-related measures.

During the landing phase, the measures showing larger between-rider differences also varied across obstacle-height conditions. At 85 cm, the larger differences involved the right hip-, right ankle-, right knee-, left hip-, and left shoulder-related measures. At 105 cm, the left hip-related measure had the largest absolute landing-phase difference (Δmean = −26.76°), accompanied by a right hip-related difference. At 140 cm, the right shoulder-related measure showed the largest absolute landing-phase difference (Δmean = −17.83°). The between-rider difference in the right ankle-related measure changed sign across conditions, from −24.58° at 85 cm to +8.18° at 105 cm and +7.08° at 140 cm.

### 3.3 Case-level kinematic profiles of the two elite riders

When obstacle-height conditions and jumping phases were considered together, the two riders showed different case-level patterns in the angle-related kinematic measures. These patterns were not uniform across conditions; instead, the measures showing larger between-rider differences varied across obstacle-height conditions and jumping phases.

Rider B showed larger values in both hip-related measures during the approach phase across all three obstacle-height conditions, as reflected by negative Δmean values for both measures. In contrast, Rider A showed larger values in the right shoulder-related measure and both knee-related measures during take-off at 140 cm.

Descriptively, the case-level differences included a consistent direction in the approach-phase hip-related measures, a sign change in the between-rider difference in the right shoulder-related measure during take-off across obstacle-height conditions, and larger absolute landing-phase hip-related differences at 85 and 105 cm than at 140 cm. In flight, the measures with larger between-rider differences changed across obstacle-height conditions, and no single angle-related measure showed the largest absolute between-rider difference across all three conditions.

### 3.4 Descriptive within-rider patterns across obstacle-height conditions

To characterize descriptive within-rider patterns across obstacle-height conditions, phase-specific mean values of the angle-related kinematic measures were summarized separately for Rider A and Rider B. Because only one successful trial was analyzed at each obstacle-height condition, these patterns should be interpreted descriptively and should not be considered evidence of stable within-rider adaptation attributable to obstacle height.

For Rider A, approach-phase mean values were generally highest at 105 cm, except for the right hip- and left ankle-related measures, which were highest at 140 cm. At take-off, bilateral shoulder- and knee-related measures showed progressively higher mean values across the 85, 105, and 140 cm conditions, while both hip-related measures showed progressively lower mean values. The ankle-related measures showed side-specific patterns: the left ankle-related measure was highest at 140 cm, whereas the right ankle-related measure was highest at 105 cm. In flight, most measures had their highest mean values at 140 cm, although the right hip- and right knee-related measures were highest at 105 cm. During landing, the right hip- and left knee-related measures decreased across the three conditions, whereas the right shoulder- and left ankle-related measures increased. The left shoulder-, right knee-, and right ankle-related measures were highest at 105 cm, while the left hip-related measure was highest at 85 cm.

For Rider B, approach-phase shoulder-, hip-, and knee-related measures were generally highest at 105 cm, whereas both ankle-related measures were highest at 140 cm. At take-off, the right shoulder-related measure decreased across the three conditions, while the left shoulder-related measure remained relatively stable and the left ankle-related measure increased. The left hip- and bilateral knee-related measures were highest at 105 cm, the right hip-related measure was highest at 140 cm, and the right ankle-related measure was highest at 85 cm. In flight, the hip-related measures, knee-related measures, and left ankle-related measure were highest at 140 cm; both shoulder-related measures were highest at 105 cm, and the right ankle-related measure was highest at 85 cm. During landing, both shoulder-related measures and the left ankle-related measure were highest at 140 cm, the hip-related measures and left knee-related measure were highest at 105 cm, and the right knee- and right ankle-related measures were highest at 85 cm.

## 4 Discussion

### 4.1 Interpretation of key angle-related differences

The two elite riders showed phase-specific case-level patterns in the angle-related kinematic measures across the three obstacle-height conditions (85, 105, and 140 cm). Between-rider differences did not follow a simple monotonic pattern across the three obstacle-height conditions. The measures showing larger between-rider differences shifted across jumping phases and obstacle-height conditions, with changes in the direction of between-rider differences observed for several measures. From a movement-variability perspective, this pattern may be considered conceptually in relation to variation in movement organization across tasks and individuals rather than as a fixed technical pattern or a simple increase in the magnitude of the angle-related measures (Cowin et al., 2022). Different obstacle-height conditions and jumping phases may be associated with different task demands on the rider, and the kinematic patterns observed in this case study may reflect different ways of organizing posture and limb position within the horse–rider system (Clayton et al., 2023). Because the present study was descriptive and did not experimentally test functional outcomes, these interpretations should be regarded as hypotheses about movement organization rather than demonstrated functional mechanisms.

During approach, the hip-related differences followed a relatively consistent direction across the three obstacle-height conditions, with Rider B showing larger values in the hip-related measures than Rider A. The ankle-related differences showed changes in direction across obstacle-height conditions. Thus, the observed approach-phase differences involved both proximal and distal body regions. This descriptive pattern can be considered alongside previous work examining rider trunk kinematics and pelvic roll and pitch during riding (Clark et al., 2022; Egenvall et al., 2022).

During take-off, the between-rider difference in the right shoulder-related measure changed direction across obstacle-height conditions, and hip-related differences also changed direction across conditions. Thus, the prominence and direction of the shoulder- and hip-related differences varied across obstacle-height conditions rather than following a single pattern across all conditions. This descriptive pattern can be considered alongside previous work examining rider kinematics and electromyographic activity across different cross-country jumping tasks (Fortier Guillaume et al., 2021).

During flight, between-rider differences were not consistently concentrated in a single angle-related measure. Instead, the body regions showing larger differences changed across obstacle-height conditions. From a movement-variability perspective, this pattern may be considered conceptually in relation to variation in body-position organization during the airborne phase rather than dominance of a single measure. In this exploratory case study, however, such variation should be interpreted as case-level kinematic variation rather than as evidence of inconsistency or functional significance.

During landing, the larger between-rider differences involved both proximal and distal body regions. Hip-related differences were more prominent at 85 and 105 cm, whereas the right shoulder-related measure showed the largest landing-phase between-rider difference at 140 cm. The between-rider difference in the right ankle-related measure also changed sign across obstacle-height conditions. Thus, the observed landing-phase differences were not limited to one body region; rather, the relative prominence of hip-, knee-, shoulder-, and ankle-related measures varied across obstacle-height conditions. Previous research has examined shock attenuation and trunk electromyographic activity during equestrian riding (Elmeua González and Šarabon, 2021); however, these phenomena were not directly measured in the present study and should not be inferred from the observed angle-related patterns alone.

### 4.2 Comparison with previous studies and conceptual grounding

The present results relate to earlier equestrian biomechanics research mainly at a conceptual level, rather than through direct variable-level correspondence with individual kinematic measures. Earlier work has examined rider position and trunk kinematics in different rider groups or measurement settings (Clark et al., 2022; Nankervis et al., 2015), rider asymmetry and horse–rider interaction (Clayton et al., 2023; MacKechnie-Guire et al., 2020; Nemecek et al., 2018), rider kinematics and electromyographic activity across different cross-country jumping tasks (Fortier Guillaume et al., 2021), and equine kinematics and muscle activity during submaximal jumping (St George et al., 2021). Compared with these studies, the present analysis focused on two elite Chinese show jumping riders under real jumping conditions and examined shoulder-, hip-, knee-, and ankle-related kinematic patterns across obstacle-height conditions and jumping phases. The observed between-rider differences were not concentrated in a single angle-related measure. Instead, measures showing larger between-rider differences shifted across body regions, obstacle-height conditions, and jumping phases.

This pattern can be considered conceptually in relation to movement-variability frameworks, although the present design did not quantify within-rider movement variability across repeated trials. Movement-variability frameworks describe how sporting movements may vary across repeated performances and under different task constraints (Cowin et al., 2022). Equestrian research has also examined coordination variability in competitive dressage riders during simulated trot, although that context differs from real show jumping (Wilkins et al., 2025). In the present case study, different obstacle-height conditions and jumping phases may involve different task demands on rider posture and limb organization. The directional changes and phase-specific shifts observed in the two riders should therefore be treated as case-level descriptive kinematic patterns rather than as evidence of a uniform technical response across all obstacle-height conditions and jumping phases.

Taken together, the redistribution of between-rider differences across jumping phases and obstacle-height conditions can be considered within a broader horse–rider systems perspective (Clayton et al., 2023; MacKechnie-Guire et al., 2020; Nemecek et al., 2018). Within this framework, rider movement organization may vary in relation to changing task demands and the ongoing movement of the horse rather than being represented by an isolated change in a single body region. The shifts in the relative prominence of shoulder-, hip-, knee-, and ankle-related measures across jumping phases and obstacle-height conditions may therefore be considered conceptually in relation to how multiple body regions are organized within the horse–rider system. This interpretation also links the present observations conceptually with research on movement variability and rider coordination, in which variation across movement components may provide information about how movement is organized under changing task constraints (Cowin et al., 2022; Wilkins et al., 2025). However, the present study did not directly quantify intersegmental coordination, horse kinematics, horse–rider interaction forces, or neuromuscular control. Therefore, the data cannot identify a specific coordination strategy or causal biomechanical mechanism, nor can they determine the direction or magnitude of horse–rider coupling. The observed patterns should instead be regarded as hypotheses for future studies combining synchronized horse–rider kinematics with time-resolved coordination analyses and kinetic or neuromuscular measurements.

Direct comparisons with previous studies should still be made cautiously. Differences in rider population, horse–rider pairing, jumping task, obstacle setting, measurement approach, and selected variables limit one-to-one comparison of individual angle-related measures. Methodological work comparing marker-based and markerless approaches in equestrian rider kinematic analysis also indicates that measurement approach should be considered when interpreting rider kinematic data (Cameron-Whytock et al., 2025). The present results should therefore be interpreted as an exploratory description of angle-related kinematic patterns across obstacle-height conditions and jumping phases in two elite Chinese riders, rather than as direct confirmation or refutation of previous research.

### 4.3 Limitations

This exploratory case study included only two elite riders, and one successful jump trial per rider was analyzed at each obstacle-height condition. Accordingly, the observed patterns cannot be assumed to represent stable rider-specific technical characteristics and should be interpreted only as case-level kinematic descriptions. Because repeated trials under identical conditions were not included in the analysis, within-rider consistency, stability, and movement repeatability could not be evaluated, and some of the observed differences may reflect trial-specific variability. The findings should therefore not be generalized to the broader population of elite show jumping riders. In addition, the obstacle heights were tested in a fixed ascending sequence (85 cm, 105 cm, and 140 cm). Consequently, potential order, familiarization, adaptation, or fatigue effects could not be separated from obstacle-height-related differences, and the observed changes across heights cannot be attributed solely to obstacle height.

Although within-phase SDs were reported descriptively, the principal comparisons relied on phase-averaged values of the angle-related measures. Successive frame-level observations within each phase were temporally correlated and therefore should not be interpreted as independent observations or independent experimental units. Accordingly, the within-phase SDs describe temporal variability within a single jump trial and should not be interpreted as between-trial variability or evidence of movement repeatability. Similarly, the phase-averaged values summarize a temporally structured movement sequence rather than a set of independent observations. Consequently, the analysis did not characterize the temporal sequencing of changes, transient peaks, or within-phase coordination dynamics among these measures. These dynamic features may contain biomechanically relevant information that is not captured by phase-level summaries. Future studies should therefore complement phase-averaged descriptors with time-normalized waveform or trajectory analyses and, where appropriate, quantitative measures of temporal coordination.

Other limitations concerned measurement conditions and horse-related context. Neither intra-rater nor inter-rater digitization reliability was quantitatively assessed, and quantitative indicators of DLT reconstruction quality, such as calibration accuracy or reconstruction error, were not independently quantified. Consequently, operator-related digitization variability and the measurement precision of the reconstructed coordinates and derived angle-related kinematic measures could not be fully evaluated. The influence of the smoothing procedure on the derived time series was not evaluated using residual or sensitivity analysis. Accordingly, relatively small Δmean values should be interpreted cautiously, because their magnitude may be influenced by unquantified digitization and reconstruction uncertainty. Because these sources of measurement uncertainty were not quantitatively characterized, the present study cannot determine whether small between-rider differences exceed the unquantified measurement uncertainty associated with the measurement process. Data were collected under field-based video measurement conditions, and the analysis focused on landmark-derived angle-related kinematic measures of the rider rather than direct anatomical joint-angle measurements. Although the same horse was used to reduce variability related to horse identity, horse kinematics, approach velocity, stride characteristics, and fatigue-related variables were not quantitatively assessed. Consequently, unmeasured within-horse variation across trials and horse–rider interaction effects may have contributed to the observed rider kinematic patterns; their potential contributions could not be independently evaluated in the present design. The interpretations should therefore be regarded as hypothesis-generating rather than as validated functional explanations, and the findings do not support generalizable training recommendations. Larger samples, repeated trials under identical conditions, independent reliability assessment, and more detailed measurement or control of horse-related variables are needed. Because the present study focused on kinematic descriptors, it could not directly characterize force production, mechanical loading, or neuromuscular control. Future studies should integrate kinematic analysis with kinetic measures, including force- or pressure-related indicators, and neuromuscular measurements to provide a more comprehensive biomechanical characterization of rider movement during show jumping. Longitudinal tracking could further clarify the stability, adaptability, and practical relevance of rider movement organization across obstacle-height conditions and jumping phases.

### 4.4 Practical implications

Given the exploratory case study design, the practical implications should be interpreted cautiously. The results do not support generalizable training recommendations or the designation of specific measures as established monitoring priorities. The observed patterns are better treated as candidate observation points for rider-specific evaluation and future validation.

The patterns observed across obstacle-height conditions and jumping phases suggest that future rider-monitoring research may benefit from considering multiple phases and measures rather than relying on a single measure. In this dataset, the approach-phase hip-related measures, the right shoulder-related measure during take-off, shifts in the body regions showing larger differences during flight, and landing-phase differences involving both proximal and distal body regions could be examined further in future validation studies.

The different case-level profiles also provide a rationale for examining individualized feedback approaches in future studies. Whether feedback informed by such case-level movement profiles improves rider technique or performance remains to be established through direct experimental validation.

These points may help shape future rider-monitoring studies. Where feasible, future studies could examine rider angle-related kinematic measures and horse-related responses together across obstacle-height conditions and jumping phases. Because the present analysis was based on two elite riders and one successful trial per rider at each obstacle-height condition, these applied considerations should be regarded as preliminary and require future validation. Larger samples, repeated trials, and longitudinal tracking are needed before generalizable training recommendations can be made.

## 5 Conclusion

This exploratory case study described angle-related kinematic patterns across obstacle-height conditions and jumping phases in two elite Chinese show jumping riders during real jumping. Between-rider kinematic differences did not show a consistent monotonic increase across obstacle-height conditions. The measures showing larger between-rider differences shifted across obstacle-height conditions and jumping phases. At the case level, the descriptive patterns highlighted in the present analysis included the relatively consistent direction of hip-related differences during approach, the change in direction of the between-rider difference in the right shoulder-related measure during take-off across obstacle-height conditions, changes in the body regions showing larger differences during flight, and landing-phase differences involving both proximal and distal body regions.

The results add case-level three-dimensional kinematic evidence for elite Chinese show jumping riders, a group for which direct quantitative rider-centered evidence remains limited. In these two riders, the observed angle-related kinematic patterns varied across obstacle-height conditions and jumping phases. Because only two riders and one successful jump trial per rider at each obstacle-height condition were analyzed, the findings should not be generalized to the broader population of elite show jumping riders or used to support generalizable training recommendations. They are better regarded as candidate observation points and testable hypotheses for future rider-specific evaluation. Larger samples, repeated trials under identical conditions, more detailed horse-related measurements, and longitudinal designs are needed to examine the stability and applied relevance of these patterns.

## Data Availability

The original contributions presented in the study are included in the article/supplementary material, further inquiries can be directed to the corresponding author.
